# Effect and Safety of Transcutaneous Auricular Vagus Nerve Stimulation on Recovery of Upper Limb Motor Function in Subacute Ischemic Stroke Patients: A Randomized Pilot Study

**DOI:** 10.1155/2020/8841752

**Published:** 2020-08-01

**Authors:** Dandong Wu, Jingxi Ma, Liping Zhang, Sanrong Wang, Botao Tan, Gongwei Jia

**Affiliations:** ^1^Department of Rehabilitation Medicine, The Second Affiliated Hospital of Chongqing Medical University, 76 Linjiang Road, Yuzhong District, Chongqing 400010, China; ^2^Department of Neurology, Chongqing General Hospital, University of Chinese Academy of Sciences, Yuzhong District, Chongqing 400013, China; ^3^Chongqing Key Laboratory of Neurodegenerative Diseases, Yuzhong District, Chongqing 400013, China; ^4^Chongqing Medical University, 1 Yixueyuan Road, Yuzhong District, Chongqing 400010, China

## Abstract

**Background:**

Transcutaneous auricular vagus nerve stimulation (taVNS) is regarded as a potential method for recovery in stroke. The effectiveness of taVNS in acute and subacute stroke should be further discussed as previously, only a few small-scale trials have focused on chronic stroke patients. The objective of this study is to investigate the effect and safety of taVNS on upper limb motor function in subacute ischemic stroke patients.

**Methods:**

Twenty-one subacute ischemia stroke patients with single upper limb motor function impairment were enrolled and randomly assigned to conventional rehabilitation training with real or sham taVNS, delivered for 15 consecutive days. Electrodes were fixed to the cymba conchae of the left ear with or without electrical stimulation. Conventional rehabilitation training was performed immediately after the end of real or sham taVNS by the same therapists. Baseline assessments were performed on day 0 of enrollment, and posttreatment evaluations were performed at 15 days, 4 weeks, and 12 weeks after the first intervention. The assessment included the upper limb Fugl-Meyer assessment (FMA-U), the Wolf motor function test (WMFT), the Functional Independence Measurement (FIM), and Brunnstrom stage. Heart rate (HR) and blood pressure (BP) were measured before and after each taVNS intervention. At the same time, any adverse effects were observed during the procedure. Outcomes were assessed by a blind evaluator.

**Results:**

There were no significant differences in FMA-U, WMFT, FIM, and Brunnstrom scores between the two groups at baseline (*P* > 0.05). At the endpoint, the FMA-U, WMFT, and FIM scores were significantly higher than before treatment (*P* < 0.05), and there was a significantly greater improvement of those measurements in taVNS group compared with sham-taVNS group (*P* < 0.05). Significant improvements in FMA-U score were found between groups at follow-up. Only one case of skin redness occurred during the study.

**Conclusions:**

This study revealed that taVNS appeared to be beneficial to the recovery of upper limb motor function in subacute ischemia stroke patients without obvious adverse effects. *Trial registration*. This trial is registered with ChiCTR1800019635 on 20 November 2018 (http://www.chictr.org.cn/showproj.aspx?proj=32961).

## 1. Introduction

Ischemia stroke refers to localized ischemic necrosis caused by blood circulation disorder in the brain, which has the characteristics of high morbidity, high mortality, and high disability [1]. Limb motor dysfunction, especially upper limb motor dysfunction, is the most common dysfunction after ischemia stroke [2]. In the clinical setting, the gross motor function of the lower limbs of patients with cerebral infarction hemiplegia recover faster, while the recovery of upper limb movements, mainly composed of flexible and coordinated skill movements, is relatively slow and difficult. Currently, for the rehabilitation of upper extremity dyskinesia, comprehensive rehabilitation programs such as neuromuscular electrical stimulation, mandatory exercise therapy, occupational therapy, imaginary therapy, and rehabilitation robot are adopted [3]. However, the implementation of the above treatment strategies still requires further clinical validation and optimization [4]. Novel and more effective treatments are needed.

Vagus nerve stimulation (VNS) refers to a method of stimulating the vagus nerve with an implantable device. The electrode is fixed on the vagus nerve by surgery, and the stimulation device is buried in the chest to stimulate the stimulator [5]. Recent clinical trials show that VNS can improve upper extremity motor function in stroke patients with upper extremity motor dysfunction [6, 7]. Transcutaneous auricular vagus nerve stimulation (taVNS) is a noninvasive method of VNS via transcutaneous stimulation of the peripheral auricular branch of the vagus nerve [8] and regarded as a potentially safer, better-tolerated method for sensory and motor recovery in chronic stroke [9, 10]. Previously, one study has reported that taVNS improves neurobehavioral recovery in acute stroke rats [11]. However, there are no clinical trials to verify the effectiveness of taVNS in acute and subacute stroke. The purpose of the study is to validate the efficacy and safety of taVNS in the recovery of upper limb motor function in subacute ischemia stroke patients through a prospective randomized controlled trial for the first time.

## 2. Methods

### 2.1. Study Design

A prospective, single-blinded, randomized controlled trial was conducted. Eligible participants were recruited and then randomly assigned to one of the following two groups: taVNS group or sham-taVNS group. A research assistant who was not involved in the evaluations or interventions performed the allocation sequence by random allocation software. This clinical trial was approved by the Institutional Review Board and Hospital Research Ethics Committee of Chongqing Medical University (approval NO. 2018208) and was conducted at the Department of Rehabilitation Medicine, the Second Affiliated Hospital between Dec 2018 and May 2019. The Chinese Clinical Trial Registry (a nonprofit organization, established according to both the WHO International Clinical Trials Register Platform Standard and Ottawa Group Standard) granted full approval of the study protocol, recruitment materials, and consent form (http://www.chictr.org.cn/showproj.aspx?proj=32961; registration no. ChiCTR1800019635). All methods were carried out in accordance with the approved ethical guidelines. After the study had been completely described to the participants, they all signed written informed consent forms. Outcome assessors were blinded to the treatment for the duration of the study.

### 2.2. Participants

Each participant was initially interviewed and evaluated by an attending physician, and the evaluation was then confirmed by another well-trained and experienced physician. The inclusion criteria were as follows: (1) first-time ischemia stroke; (2) in the acute or subacute phase of stroke (between 0.5 and 3 months postonset); (3) single upper limb motor function impairment; and (4) ability to follow instructions, with no obvious cognitive impairment. The exclusion criteria were as follows: (1) hemorrhagic stroke, which lead to the heterogeneity of lesion etiology; (2) advanced cardiac, pulmonary, liver, kidney dysfunction or blood system diseases; (3) malignant tumors or infectious diseases; (4) other neurologic or musculoskeletal diseases that could interfere with the assessments of this study; (5) low heart rate (<60 bpm); (6) previous surgical intervention on the vagus nerve; and (7) Botox injections or any other nonstudy active rehabilitation of the upper extremity 12 weeks prior to or during therapy.

### 2.3. Interventions

**Figure 1:**
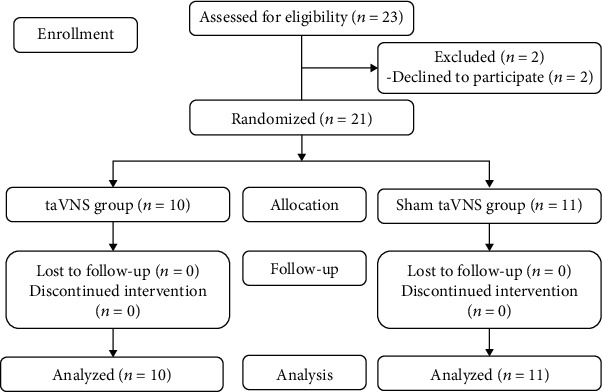
Flow diagram of the selection process in the study.

**Figure 2:**
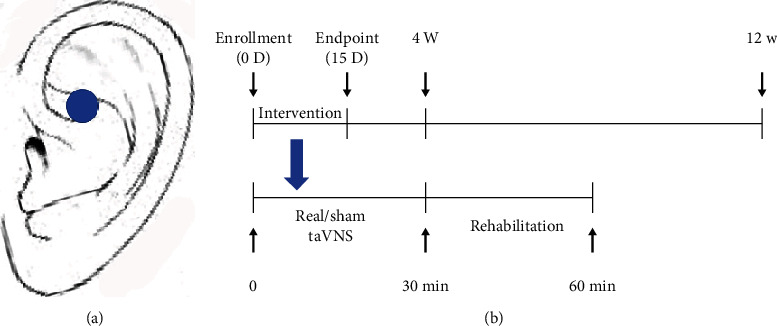
(a) Position of the taVNS stimulation (cymba conchae). (b) Experimental timeline. D: day; W: week; min: minute.

A flow chart of the trial selection process was shown in Figure 1. All patients were questioned about their age, gender, duration of onset, stroke location, and hemiplegic side. taVNS was applied by a BHD-1A transcutaneous electrical stimulation therapy instrument (Bohua, Weihai, China). The left auricular branch vagus nerve was stimulated by the modified dot-like electrodes that were fitted to the cymba conchae (Figure 2(a)). The parameters were selected as follows: 600 pulses (intratrain pulse frequency = 20 Hz; pulse duration = 0.3 ms), lasting 30 seconds each time, stimulating once every 5 minutes [12]. Intensity of taVNS was individually selected by the patients according to tolerance. The participant could withdraw from the trial if he could not tolerate the stimulation. Stimulation was performed for 30 minutes per day for 15 consecutive days. In the sham-taVNS group, electrodes were fixed to the cymba conchae of the left ear without electrical stimulation. Conventional rehabilitation training was customized and applied to the limbs and the trunk with the patient according to their capacity. Conventional rehabilitation training involved postural control, proprioception exercises, neuromuscular facilitation, gait training, and always at the upper limit of their capacity. For example, flexion and extension of the elbow were trained repetitively until the patient reported a sensation of fatigue, against an adapted resistance by the therapist. All techniques were allowed for these trainings. Rehabilitation training, lasting approximately 30 minutes, was performed immediately after the end of real or sham taVNS by the same therapists for patients in both groups to prevent bias that could be introduced by the personality of therapists (Figure 2(b)).

### 2.4. Outcome Measures

Upper limb Fugl-Meyer assessment (FMA-U) [13], Wolf motor function test (WMFT) [14], Functional Independence Measurement (FIM) [15], and upper limb Brunnstrom stage [16] were measured for assessing the effect. These assessments were performed at baseline and at the end of the intervention. To observe long-term effects, FMA-U was evaluated at 4 weeks and 12 weeks after intervention. FMA-U was used to evaluate upper extremity motor function after stroke in 33 items on a 3-point scale (maximum motor score, 66 points) [13]. WMFT uses a 6-point scale that ranges from 0 to 5 (normal) for a maximum score of 75 for the 15 tasks that are used to assess upper extremity motor function after stoke [14]. FIM measures independence in basic activities of daily living and its score ranges from 18 (maximum level of dependence) to 126 (highest level of independence) [15]. Brunnstrom staging is a six-stage evaluation that models the motor recovery process following stroke-induced hemiplegia (stages 1–6, 1: no activity; 2: spasticity appears; 3: spasticity is prominent; 4: patient begins to activate muscles selectively outside the flexor and extensor synergies; 5: spasticity decreases; 6: isolated movements in smooth, well-coordinated manner) [16].

The main safety outcome measure was the number of serious adverse events related to the device or therapy, such as skin toxicity (pain, skin erythema, burns, etc.), hoarseness, and dysphagia. Heart rate (HR) and blood pressure (BP) were also observed before and after each intervention.

### 2.5. Sample Size and Statistical Analysis

Because this was a pilot study, no formal sample size calculation was calculated. Analyses were performed on the intention-to-treat population. SPSS 20.0 statistical software was used to analyze the data. The measurement data were expressed as means ± SDs (standard deviation). For comparisons of baseline characteristics, the Chi-square test was used for categorical variables, and the Kruskal-Wallis test was used for continuous variables. Prior to comparisons, we tested whether the data were normally distributed and the variances were equal. If so, paired *t*-test was used to compare the change of measurement data from baseline to postintervention and follow-up in each group. Independent sample *t*-test was used for comparisons of the change in outcome measures between groups. If the data were not normally distributed nonparametric, Wilcoxon and Mann-Whitney *U* tests were applied. A mixed-model repeated ANOVA with pre-post data, days as within subject factors, and groups as between subject factors was performed for HR and BP measures. *P* value < 0.05 was considered to be statistically significant.

## 3. Results

### 3.1. Patient Characteristics

Twenty-one patients consented to participate in the study and were randomized to taVNS groups (10 patients) or sham-taVNS groups (11 patients). No patients aborted the study for any reasons. There were no significant differences between taVNS groups and sham-taVNS groups with respect to age, gender, duration of onset, hemiplegic side, systolic blood pressure (SBP), diastolic blood pressure (DBP), and heart rate (HR) (*P* > 0.05, Table 1).

### 3.2. Outcomes

#### 3.2.1. Effect

There were no significant differences between taVNS group and sham-taVNS group with respect to FMA-U, WMFT, FIM, and Brunnstrom scores at baseline (*P* < 0.05). There were significant differences between the taVNS group and sham-taVNS group on most measures (*P* = 0.024, *P* = 0.036, and *P* = 0.013) except for Brunnstrom stage (*P* = 0.857) at endpoint. All measures improved from baseline after interventions (*P* < 0.05). The improvement in FMA-U, WMFT, and FIM scores in the taVNS group was significantly greater than in sham-taVNS group after 15 days of intervention (*P* ≤ 0.001, *P* ≤ 0.001, and *P* = 0.034). No difference was found between the two groups with regard to a change of Brunnstrom stage (*P* = 0.831, Table 2).

### 3.2.2. Long-Term Effect

FMA-U scores remained significantly higher at the 4-week and 12-week follow-up after first intervention compared with baseline in both groups, and a significantly greater improvement was evident in taVNS group compared with the sham-taVNS group (*P* < 0.05, Table 3).

### 3.2.3. Adverse Events

Only one adverse event was noted during the whole procedure. One patient in the taVNS group developed skin redness at the point of contact of the auricle skin electrodes after the third treatment, which returned to normal 6 hours later. There were no unpleasant sensations or other discomforts.

**Figure 3:**
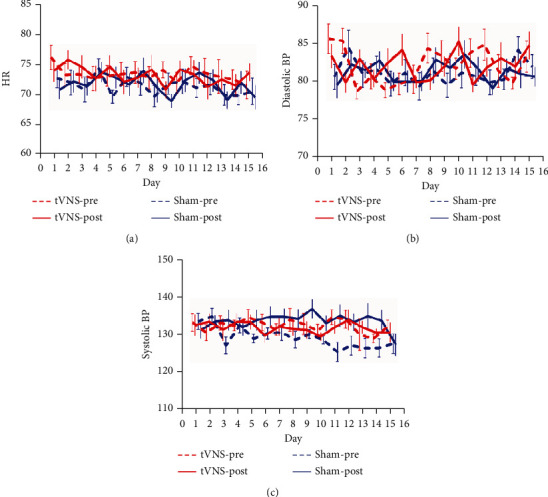
The effects of taVNS on HR and BP. BP: blood pressure; HR: heart rate.

No significant pre-post differences (*F*(1.19) = 0.028, *P* = 0.868) nor group differences (*F*(1.19) = 0.311, *P* = 0.584) were found for HR (Figure 3(a)). The same main effects for diastolic blood pressure (DBP) (*F*(1.19) = 0.014, *P* = 0.908; *F*(1.19) = 0.015, *P* = 0.903; Figure 3(b)) were exhibited. No significant group differences were found for systolic blood pressure (SBP) (*F*(1.19) = 0.131, *P* = 0.722), while a significant pre-post ∗ group interaction (*F*(1.19) = 14.344, *P* = 0.01) related to pre-post differences (*F*(1.19) = 8.097, *P* = 0.01) was evident. The change in SBP was mild (-0.607 mmHg in the taVNS group and 4.273 mmHg in the sham-taVNS group, Figure 3(c)).

## 4. Discussion

The primary objective of this blinded randomized pilot study was to investigate the effects and safety of taVNS on upper limb motor function in subacute ischemic stroke patients. Significantly greater improvements were found in FMA-U, WMFT, and FIM scores in the taVNS group at endpoint. Meanwhile, a significantly greater improvement of FMA-U score was evident in the taVNS group compared with the sham-taVNS group at 4 weeks and 12 weeks. Only one case of an adverse event that was related to the contact of the auricle skin electrodes was noted. Vagal innervation was important to cardiac function [17]. In order to identify any potential cardiovascular harm, we monitored HR and BP during treatment sessions. Our data showed no clinically significant change in cardiovascular parameters throughout the treatment sessions.

Initial clinical trials of VNS occurred between 1988 and 1995 in patients with refractory epilepsy and showed that VNS was safe and well tolerated [18]. VNS has been researched as a potential treatment in many neurological disorders such as migraine [19], traumatic brain injury [20], chronic tinnitus [21], Alzheimer's disease [22], Parkinson's disease [23], intracerebral hemorrhage [24], and even ischemic stroke [7]. In animal studies, VNS had been reported to attenuate cerebral infarct volume, reduce neurological deficits, and improve forelimb function [25, 26]. Since Dawson et al. [6] performed the first-in-human evaluation of VNS paired with upper-limb rehabilitation after ischemic stroke, few clinical studies have focused on the efficacy and safety of VNS on stroke patients [7, 27]. In fact, complications and the failure of VNS therapy were not rare in these studies. For example, laryngopharyngeal dysfunction (hoarseness, dyspnea, and coughing) occurred in about 66% of patients but was usually transitory and due to the inferior (recurrent) laryngeal nerve that was stimulated and also related to the frequency of stimulation [28]. Surgery for revision of VNS accounted for about 50% of cases [29].

Noninvasive VNS was safe and well-tolerated, and adverse events were very rare [30]. There are two types of noninvasive VNS: taVNS that stimulates at the external ear (an auricular branch of the vagus nerve) and tcVNS that stimulates at the cervical region (a cervical branch of the vagus nerve) [31]. The cymba conchae is the most effective and optimal location for taVNS therapy that was demonstrated by functional magnetic resonance imaging (fMRI) [32]. The central projections of the auricular branch of the vagus nerve were consistent with the “classical” central vagal projections and could be accessed noninvasively via the external ear [33]. taVNS is found to activate brainstem afferent vagal nuclei in stroke rats [8] and the motor cortex, insula, the precentral gyrus, and the thalamus in healthy participants [34, 35]. These results indicated that taVNS may share the same mechanism or pathway as VNS.

In our study, rehabilitative training was performed immediately after the end of taVNS. VNS reinforced the effects of rehabilitative training to improve recovery of motor function [36, 37]. The timing of VNS-rehabilitation coupling was essential because enhancement of VNS has not shown to be effective when VNS followed rehabilitation [37, 38]. Chronic ischemic stroke rats underwent rehabilitative training with VNS, rehabilitative training with VNS delivered 2 hours after daily rehabilitative training, and rehabilitative training without VNS. The study showed that subjects in the Paired VNS group displayed a 85.9% ± 6.1% recovery of forelimb strength, subjects in the Delayed VNS group exhibited a 42.1% ± 8.0% recovery of forelimb strength, while subjects in the Rehab group displayed a 47.2% ± 13.4% recovery of forelimb strength in the last week of rehabilitative training [37]. This result supported the view that the synergistic effect of VNS and rehabilitation depended on neuroplasticity, a time-dependent phenomenon. Our study indicated that taVNS had a promoting effect on the recovery of upper limb motor function in subacute stroke patients. FMA-U scores improved in two groups after the intervention but the change of FMA-U was significantly higher in the taVNS group than in the sham-taVNS group (6.9 versus 3.18; *P* ≤ 0.001) at endpoint, and a similar change was found at 4 weeks (7.7 versus 3.36; *P* ≤ 0.001) and at 12 weeks (7.48 versus 4.18; *P* = 0.038) after the first intervention. FMA-U was deemed a core outcome in stroke recovery [39], and this change was considered clinically significant in stroke patients [40]. Indeed, a similar change of FMA-U was found in other clinic trials [6, 7, 10, 12]. Interestingly, the changes from FMA-U are larger in implanted VNS trials [6, 7]. In acute ischemia stroke animals, VNS resulted in a greater reduction of infarct volume compared to taVNS (50% versus 28%) [8, 26]. Weaker activation of the central vagal pathway by taVNS as compared to VNS was proven [8], and this may be the reason for the reduced efficacy in animal and human studies with taVNS compared to VNS. The significantly greater improvement of WMFT and FIM scores in our study also indicated that taVNS had a promoting effect on the recovery of upper limb motor function in subacute stroke patients. But there was no difference between the two groups in respect to a change in Brunnstrom stage. The reason may be that the distinction of the Brunnstrom stage scale is too small. There are only six grades in the recovery stage of motor function in the Brunnstrom evaluation. And not all patients will recover according to these stages; some patients may skip some stages in the recovery process [16]. Our study observed that the change of FMA-U in the sham-taVNS group increased from 3.36 points at 4 weeks to 4.18 points at 12 weeks while the change of FMA-U in the taVNS group decreased from 7.70 points at 4 weeks to 7.40 points at 12 weeks. Studies verified that neurologic and functional recovery show faster recovery in the first weeks poststroke because of spontaneous neurologic recovery [41, 42]. The decrease in the change of FMA-U in the taVNS group implied that the promoting effect of taVNS may weaken with time after the end of intervention in subacute stroke patients. One preclinical study even found the benefits of VNS performed at 7 days after ischemia could be maintained for up to 7 weeks after the end of stimulation [36]. In fact, the potential mechanisms of VNS were not exactly the same according to different stroke stages and remained to be determined. In chronic stroke rats, VNS does not reduce lesion size but supports recovery by promoting neuroplasticity [37], while in acute stroke rats, neuroprotection, neurogenesis, and neuroplasticity were regarded as the potential mechanisms of VNS [36, 43–45]. The duration of benefits from VNS should be researched in further studies in acute and chronic stroke patient. Our study showed that taVNS was safe. Mean stimulation intensity was 1.66 mA, and no patient was required to stop stimulation. Only one skin redness case that should be related to contact between the electrode and the skin was noted. Our data showed no clinically significant change in cardiovascular parameters. A slight and asymptomatic reduction of SBP was found in the taVNS group. The effects of taVNS on cardiovascular parameters (HR and BP) were inconsistent in human studies. A significantly decreased HR and systolic BP was found in taVNS-treated coronary artery disease patients [46], while no significant change in HR and BP was found in stroke patients treated by taVNS with robotic rehabilitation [12] and among healthy volunteers [35]. Thick afferent A beta myelinated axons have been consistently shown to mediate the effects of VNS [47], while the number of A beta myelinated axons varied widely between individuals [48], which may help to explain why cardiovascular parameters were inconsistent in human taVNS studies. The effects of taVNS on cardiovascular parameters may also depend on sympathetic nerve activity based on the different cardiovascular responses of taVNS in patients with different diseases.

This study has a number of limitations. First, the sample was small and limited to the early subacute phase of stroke patients [49], so the results cannot be generalized to a broader stroke population. The underlying complexity of spontaneous recovery and stroke heterogeneity were more evident in acute and subacute ischemia stroke patients, and a greater number of patients may be required for further studies. Second, the sham group in our study did not receive electrical stimulation. This configuration was the weakest version of a sham control, because it was highly likely that the electrical stimulation was perceptible in the active taVNS group, while the sham group would not perceive any sensation given the absence of electrical stimulation. Therefore, we could not blind our participant in this study. The earlobe was the most widely used sham stimulation site in previous taVNS studies, even though it is not physiologically inert [32]. Third, open-loop taVNS was applied in our study. Closed-loop VNS/taVNS was applied in most of the earlier stroke clinical trials [6, 10]. Closed-loop neuromodulation had been shown to be clinically more effective than open-loop neuromodulation [50], even though open-loop VNS can activate brainstem afferent vagal nuclei and reduce infarct volume in rats [8]. Closed-loop taVNS should be researched in future studies. Fourth, the ideal stimulation parameters for taVNS were one of the most critical challenges for its application, as these parameters had enormous impacts on clinical efficacy. In fact, the optimum timing of VNS initiation and numerous best parameters such as stimulation sites and sides, electrode and waveform configuration, efferent or afferent stimulation, and titration protocols are not known. Our treatment parameters may have an impact on the outcome of treatment. It is necessary to discuss the treatment parameters in future studies.

## 5. Conclusions

The results from this study demonstrated that taVNS appeared to be beneficial to the recovery of upper limb motor function in subacute ischemia stroke patients and without obvious adverse effects. Future studies are needed to confirm the optimum timing and ideal stimulation parameters and unveil the mechanisms of action of this innovative approach. It is necessary to further confirm the efficacy of taVNS for the chronic phase and acute phase of stroke in large sample, randomized, controlled clinical trials.

## Figures and Tables

**Table 1:** Baseline characteristics.

Variable	taVNS groups	Sham-taVNS groups	*P* value
*n*	10	11	
Age (y)	64.50 ± 9.97	61.82 ± 10.63	0.559
Gender, m/f	5/5	8/3	0.284
Duration of onset (d)	36.30 ± 9.23	35.55 ± 6.47	0.829
Hemiplegic side, r/l	6/4	8/3	0.537
Stroke location			
MCA	9	9	
ACA	0	0	
PCA	0	0	
MCA+ACA	1	2	
SBP (mmHg)	130.2 ± 11.09	127.5 ± 10.77	0.572
DBP (mmHg)	78.4 ± 9.65	76.73 ± 9.35	0.691
HR	75.10 ± 6.35	72.64 ± 5.43	0.350
Intensity (mA)	1.66 ± 0.40		

Data expressed as mean ± SD. y: year; m: male; f: female; d: day; r: right; l: left; MCA: middle cerebral artery; ACA: anterior cerebral artery; PCA: posterior cerebral artery; SBP: systolic blood pressure; DBP: diastolic blood pressure; HR: heart rate.

**Table 2:** Outcome mean differences in change from baseline.

Variable	Baseline		Endpoint		Between-group difference	
	taVNS group	Sham-taVNS group	taVNS group	Sham-taVNS group	Mean^∗^ (95% CI)	*P*
*n*	10	11	10	11		
FMA-U	17.50 ± 4.91	16.82 ± 3.89	24.40 ± 4.62	20.00 ± 3.58		0.024
FMA-U mean change from baseline (95% CI)			6.90 ± 1.85 (5.57 to 8.23)	3.18 ± 1.17 (2.40 to 3.97)	-3.72 (-5.12 to -2.32)	≤0.001
WMFT	20.60 ± 4.62	20.45 ± 3.93	27.10 ± 3.81	23.36 ± 3.78		0.036
WMFT mean change from baseline (95% CI)			6.50 ± 2.37 (4.81 to 8.20)	2.91 ± 1.14 (2.15 to 3.67)	-3.59 (-5.27 to -1.92)	≤0.001
FIM	91.80 ± 6.03	88.82 ± 6.13	102.3 ± 5.77	95.45 ± 5.72		0.013
FIM mean change from baseline (95% CI)			10.50 ± 4.93 (6.98 to 14.02)	6.64 ± 2.58 (4.90 to 8.37)	-3.86 (-7.41 to -0.32)	0.034
Brunnstrom	2.50 ± 1.08	2.64 ± 0.81	3.20 ± 0.92	3.27 ± 0.90		0.857
Brunnstrom mean change from baseline (95% CI)			0.70 ± 0.67 (0.22 to 1.18)	0.64 ± 0.67 (0.18 to 1.09)	-0.06 (-0.68 to 0.55)	0.831

^∗^Mean difference between groups in change from baseline scores. Data expressed as mean ± SD. FMA-U: upper limb Fugl-Meyer assessment; WMFT: Wolf motor function test; FIM: Functional Independence Measurement.

**Table 3:** FMA-U score at 4 and 12 weeks after first intervention.

FMA-U	taVNS group	Within-group (*P* value)	Sham-taVNS group	Within-group (*P* value)	Between-group (*P* value)
Baseline	17.50 ± 4.91		16.82 ± 3.89		
4 w	24.90 ± 4.43	≤0.001	20.18 ± 3.22	≤0.001	
12 w	25.50 ± 4.74	≤0.001	21.00 ± 3.82	0.008	
Baseline to 4 w	7.70 ± 1.49		3.36 ± 1.75		≤0.001
Baseline to 12 w	7.40 ± 1.78		4.18 ± 4.24		0.038

Data expressed as mean ± SD. FMA-U: upper limb Fugl-Meyer assessment; w: week.

## Data Availability

All data generated or analyzed during this study are included in this published article.

## References

[1] Collaborators GBDM (2017). Global, regional, and national under-5 mortality, adult mortality, age-specific mortality, and life expectancy, 1970–2016: a systematic analysis for the Global Burden of Disease Study 2016. *The Lancet*.

[2] Nakayama H., Jørgensen H. S., Raaschou H. O., Olsen T. S. (1994). Recovery of upper extremity function in stroke patients: the Copenhagen Stroke Study. *Archives of Physical Medicine and Rehabilitation*.

[3] Drummond A., Wade D. T. (2014). National Institute for Health and Care Excellence stroke rehabilitation guidance - is it useful, usable, and based on best evidence?. *Clinical Rehabilitation*.

[4] Coupar F., Pollock A., Rowe P., Weir C., Langhorne P. (2012). Predictors of upper limb recovery after stroke: a systematic review and meta-analysis. *Clinical Rehabilitation*.

[5] Coughlin M. K. (2001). Long-term treatment with vagus nerve stimulation in patients with refractory epilepsy. *AORN Journal*.

[6] Dawson J., Pierce D., Dixit A. (2016). Safety, feasibility, and efficacy of vagus nerve stimulation paired with upper-limb rehabilitation after ischemic stroke. *Stroke*.

[7] Kimberley T. J., Pierce D., Prudente C. N. (2018). Vagus nerve stimulation paired with upper limb rehabilitation after chronic stroke. *Stroke*.

[8] Ay I., Napadow V., Ay H. (2015). Electrical stimulation of the vagus nerve dermatome in the external ear is protective in rat cerebral ischemia. *Brain Stimulation*.

[9] Baig S. S., Falidas K., Laud P. J. (2019). Transcutaneous auricular vagus nerve stimulation with upper limb repetitive task practice may improve sensory recovery in chronic stroke. *Journal of Stroke and Cerebrovascular Diseases*.

[10] Redgrave J. N., Moore L., Oyekunle T. (2018). Transcutaneous auricular vagus nerve stimulation with concurrent upper limb repetitive task practice for poststroke motor recovery: a pilot study. *Journal of Stroke and Cerebrovascular Diseases*.

[11] Ma J., Zhang L., He G., Tan X., Jin X., Li C. (2016). Transcutaneous auricular vagus nerve stimulation regulates expression of growth differentiation factor 11 and activin-like kinase 5 in cerebral ischemia/reperfusion rats. *Journal of the Neurological Sciences*.

[12] Capone F., Miccinilli S., Pellegrino G., Zollo L. (2017). Transcutaneous vagus nerve stimulation combined with robotic rehabilitation improves upper limb function after stroke. *Neural Plasticity*.

[13] Hsieh Y. W., Hsueh I. P., Chou Y. T., Sheu C. F., Hsieh C. L., Kwakkel G. (2007). Development and validation of a short form of the Fugl-Meyer motor scale in patients with stroke. *Stroke*.

[14] Berardi A., Dhrami L., Tofani M., Valente D., Sansoni J., Galeoto G. (2018). Cross-cultural adaptation and validation in the Italian population of the wolf motor function test in patients with stroke. *Functional Neurology*.

[15] Dodds T. A., Martin D. P., Stolov W. C., Deyo R. A. (1993). A validation of the functional independence measurement and its performance among rehabilitation inpatients. *Archives of Physical Medicine and Rehabilitation*.

[16] Brunnstrom S. (1966). Motor testing procedures in hemiplegia: based on sequential recovery stages. *Physical Therapy*.

[17] Coote J. H. (2013). Myths and realities of the cardiac vagus. *The Journal of Physiology*.

[18] Morris G. L., Mueller W. M. (1999). Long-term treatment with vagus nerve stimulation in patients with refractory epilepsy. The Vagus Nerve Stimulation Study Group E01-E05. *Neurology*.

[19] Tassorelli C., Grazzi L., de Tommaso M. (2018). Noninvasive vagus nerve stimulation as acute therapy for migraine: the randomized PRESTO study. *Neurology*.

[20] Pruitt D. T., Schmid A. N., Kim L. J. (2016). Vagus nerve stimulation delivered with motor training enhances recovery of function after traumatic brain injury. *Journal of Neurotrauma*.

[21] Tyler R., Cacace A., Stocking C. (2017). Vagus nerve stimulation paired with tones for the treatment of tinnitus: a prospective randomized double-blind controlled pilot study in humans. *Scientific Reports*.

[22] Merrill C. A., Jonsson M. A., Minthon L., Ejnell H. (2006). Vagus nerve stimulation in patients with Alzheimer's disease. *The Journal of Clinical Psychiatry*.

[23] Farrand A. Q., Helke K. L., Gregory R. A., Gooz M., Hinson V. K., Boger H. A. (2017). Vagus nerve stimulation improves locomotion and neuronal populations in a model of Parkinson's disease. *Brain Stimulation*.

[24] Welling L. C., Welling M. S., Teixeira M. J., Figueiredo E. G. (2016). Intracerebral hemorrhage, vagus nerve stimulation, and anti-inflammatory response. *World Neurosurgery*.

[25] Ay I., Nasser R., Simon B., Ay H. (2016). Transcutaneous cervical vagus nerve stimulation ameliorates acute ischemic injury in rats. *Brain Stimulation*.

[26] Ay I., Lu J., Ay H., Gregory Sorensen A. (2009). Vagus nerve stimulation reduces infarct size in rat focal cerebral ischemia. *Neuroscience Letters*.

[27] Kilgard M. P., Rennaker R. L., Alexander J., Dawson J. (2018). Vagus nerve stimulation paired with tactile training improved sensory function in a chronic stroke patient. *NeuroRehabilitation*.

[28] Giordano F., Zicca A., Barba C. (2017). Vagus nerve stimulation: surgical technique of implantation and revision and related morbidity. *Epilepsia*.

[29] Couch J. D., Gilman A. M., Doyle W. K. (2016). Long-term expectations of vagus nerve stimulation: a look at battery replacement and revision surgery. *Neurosurgery*.

[30] Redgrave J., Day D., Leung H. (2018). Safety and tolerability of transcutaneous vagus nerve stimulation in humans; a systematic review. *Brain Stimulation*.

[31] Gurel N. Z., Huang M., Wittbrodt M. T. (2020). Quantifying acute physiological biomarkers of transcutaneous cervical vagal nerve stimulation in the context of psychological stress. *Brain Stimulation*.

[32] Yakunina N., Kim S. S., Nam E. C. (2017). Optimization of transcutaneous vagus nerve stimulation using functional MRI. *Neuromodulation*.

[33] Frangos E., Ellrich J., Komisaruk B. R. (2015). Non-invasive access to the vagus nerve central projections via electrical stimulation of the external ear: fMRI evidence in humans. *Brain Stimulation*.

[34] Kraus T., Hösl K., Kiess O., Schanze A., Kornhuber J., Forster C. (2007). BOLD fMRI deactivation of limbic and temporal brain structures and mood enhancing effect by transcutaneous vagus nerve stimulation. *Journal of Neural Transmission*.

[35] Capone F., Assenza G., Di Pino G. (2015). The effect of transcutaneous vagus nerve stimulation on cortical excitability. *Journal of Neural Transmission*.

[36] Meyers E. C., Solorzano B. R., James J. (2018). Vagus nerve stimulation enhances stable plasticity and generalization of stroke recovery. *Stroke*.

[37] Khodaparast N., Kilgard M. P., Casavant R. (2016). Vagus nerve stimulation during rehabilitative training improves forelimb recovery after chronic ischemic stroke in rats. *Neurorehabilitation and Neural Repair*.

[38] Hays S. A., Khodaparast N., Ruiz A. (2014). The timing and amount of vagus nerve stimulation during rehabilitative training affect poststroke recovery of forelimb strength. *Neuroreport*.

[39] Kwakkel G., Lannin N. A., Borschmann K. (2017). standardized measurement of sensorimotor recovery in stroke trials: consensus-based core recommendations from the stroke recovery and rehabilitation roundtable. *International Journal of Stroke*.

[40] Page S. J., Fulk G. D., Boyne P. (2012). Clinically important differences for the upper-extremity Fugl-Meyer Scale in people with minimal to moderate impairment due to chronic stroke. *Physical Therapy*.

[41] Newman M. (1972). The process of recovery after hemiplegia. *Stroke*.

[42] Gresham G. E. (1986). Stroke outcome research. *Stroke*.

[43] Jiang Y., Li L., Tan X., Liu B., Zhang Y., Li C. (2015). miR-210 mediates vagus nerve stimulation-induced antioxidant stress and anti-apoptosis reactions following cerebral ischemia/reperfusion injury in rats. *Journal of Neurochemistry*.

[44] Yang Y., Yang L. Y., Orban L. (2018). Non-invasive vagus nerve stimulation reduces blood-brain barrier disruption in a rat model of ischemic stroke. *Brain Stimulation*.

[45] Zhang L., Ma J., Jin X., Jia G., Ying J., Li C. (2017). L-PGDS mediates Vagus nerve stimulation-induced neuroprotection in a rat model of ischemic stroke by suppressing the apoptotic response. *Neurochemical Research*.

[46] Zamotrinsky A. V., Kondratiev B., de Jong J. W. (2001). Vagal neurostimulation in patients with coronary artery disease. *Autonomic Neuroscience : Basic & Clinical*.

[47] Evans M. S., Verma-Ahuja S., Naritoku D. K., Espinosa J. A. (2004). Intraoperative human vagus nerve compound action potentials. *Acta Neurologica Scandinavica*.

[48] Safi S., Ellrich J., Neuhuber W. (2016). Myelinated axons in the auricular branch of the human vagus nerve. *Anatomical Record*.

[49] Bernhardt J., Hayward K. S., Kwakkel G. (2016). Agreed definitions and a shared vision for new standards in stroke recovery research: the stroke recovery and rehabilitation roundtable taskforce. *International Journal of Stroke*.

[50] Sun F. T., Morrell M. J. (2014). Closed-loop neurostimulation: the clinical experience. *Neurotherapeutics*.

